# The structure of *Synechococcus elongatus* enolase reveals key aspects of phosphoenolpyruvate binding

**DOI:** 10.1107/S2053230X22003612

**Published:** 2022-04-03

**Authors:** Javier M. González, Ricardo Martí-Arbona, Julian C.-H. Chen, Clifford J. Unkefer

**Affiliations:** a Instituto de Bionanotecnología del NOA (INBIONATEC–CONICET), Universidad Nacional de Santiago del Estero (UNSE), RN9, Km1125, G4206XCP Santiago del Estero, Argentina; b Bioscience Division, Los Alamos National Laboratory, Los Alamos, NM 87545, USA

**Keywords:** enolases, *Synechococcus elongatus*, cyanobacteria, phosphoenolpyruvate

## Abstract

A structure–function characterization of *Synechococcus elongatus* enolase (*Se*EN) is presented, representing the first structural report on a cyanobacterial enolase. X-ray crystal structures of *Se*EN in its apoenzyme form and in complex with phosphoenolpyruvate are reported at 2.05 and 2.30 Å resolution, respectively. *Se*EN displays the typical fold of enolases, with a conformationally flexible loop that closes the active site upon substrate binding, assisted by two metal ions that stabilize the negatively charged groups. The enzyme exhibits a catalytic efficiency of 1.2 × 10^5^ *M* ^−1^ s^−1^ for the dehydration of 2-phospho-d-glycerate, which is comparable to the kinetic parameters of related enzymes. These results expand the understanding of the biophysical features of these enzymes, broadening the toolbox for metabolic engineering applications.

## Abstract 2

The first structural report of a cyanobacterial enolase is presented, disclosing an octameric arrangement and key substrate and metal-binding features.

## 1. Introduction

Photosynthetic unicellular microorganisms have been proposed as convenient, cost-effective, carbon-neutral sources for biomass production (Udayan *et al.*, 2022 ▸; Alishah Aratboni *et al.*, 2019 ▸). Microbial factories based on algae or cyanobacteria offer several advantages over biomass production from higher plants, including the following: (i) microbes may be cultured in pools located in arid uncultivable lands, (ii) microbes exhibit fast growth rates, (iii) unlike lignocellulosic biomass from plants, microbes do not require harsh and costly extraction processes, and (iv) microbes can be genetically engineered with relative ease in order to drive biomass production towards value-added chemicals (Gong *et al.*, 2017 ▸; Claassens *et al.*, 2016 ▸; Scott *et al.*, 2010 ▸). Consequently, a comprehensive understanding of cyanobacterial metabolism, including structural and functional aspects of key enzymes, is required in order to take full advantage of synthetic metabolism for biomass production. Here, we present a structural and functional study of enolase from the cyanobacterium *Synechococcus elongatus* PCC 7942, a workhorse cyanobacterial strain for engineering of photoautotrophic metabolism (Kanno & Atsumi, 2017 ▸; Lan *et al.*, 2015 ▸). Enolases catalyse the reversible dehydration of 2-phospho-d-glycerate (2PGA) into phosphoenolpyruvate (PEP), an energy-rich phosphoester central to several bioenergetic and biosynthetic metabolic pathways. We show that *S. elongatus* enolase (*Se*EN) displays a typical catalytic efficiency of the order of 10^5^ *M* ^−1^ s^−1^, as well as a characteristic three-dimensional fold where a flexible loop closes the active site following substrate binding, ensuring the proper environment for catalysis. These results broaden our understanding of the structure and function of metabolic enzymes available for metabolic engineering applications.

## 2. Materials and methods

### 2.1. Gene cloning, expression and protein purification

All chemicals and coupling enzymes were obtained from Sigma–Aldrich unless otherwise stated. The oligonucleotide-synthesis and DNA-sequencing reactions were performed by Genewiz Inc. The pET-42a(+) expression vector was purchased from Novagen. *Escherichia coli* Arctic Express (DE3) competent cells were acquired from Stratagene. *Se*EN was synthesized and subcloned into pET-42a(+) by Genewiz Inc. with codon usage optimized for protein expression in *E. coli*. The NdeI and XhoI cloning sites were used to obtain a clone expressing *Se*EN with an 8×His tag at the C-terminus of the protein (Table 1 ▸). The expression vector encoding *Se*EN was transformed into *E. coli* Arctic Express (DE3) competent cells and cultured in liquid LB or LB–agar plates supplemented with 50 µg ml^–1^ kanamycin. A single colony was cultured overnight (∼16 h) in 25 ml LB with 50 µg ml^−1^ kanamycin, and 20 ml was then used to inoculate 2 l of the same medium. Cell cultures were grown at 37°C under shaking to an OD_600 nm_ of ∼0.6, after which the temperature was decreased to 16°C and induction was initiated by the addition of isopropyl β-d-1-thiogalactopyranoside to a final concentration of 1.0 m*M*. The culture was then incubated overnight at 16°C. The bacterial cells were harvested by centrifugation at 7000*g* for 10 min at 4°C. The pellet was washed and resuspended in 20 m*M* sodium phosphate, 50 m*M* NaCl, 5 m*M* imidazole pH 7.4 (buffer *A*). 5 µg ml^−1^ DNAse and 0.1 mg ml^−1^ phenylmethylsulfonyl fluoride protease inhibitor were added to buffer *A* before cell disruption by sonication. The soluble protein was separated from the cell debris by centrifugation at 12 000*g* for 30 min at 4°C and then loaded onto two 5 ml HisTrap columns connected in tandem and equilibrated with buffer *A*. *Se*EN was eluted with a linear gradient of buffer *A* and 50% buffer *B* in ten column volumes, where buffer *B* comprises buffer *A* plus 500 m*M* imidazole. Fractions containing *Se*EN were pooled, concentrated, loaded onto a HiLoad 26/60 Superdex 200 preparative-grade gel-filtration column (GE Healthcare) and eluted with 20 m*M* HEPES, 50 m*M* NaCl pH 7.2 (buffer *C*). The purity of the protein during the isolation procedure was checked by SDS–PAGE using Kaleidoscope pre-stained standards (molecular weight 7.6–216 kDa; Bio-Rad).

### 2.2. Protein molecular-weight estimation

A Tricorn 5/150 column (GE Healthcare) was filled with Superdex 200 prep-grade resin and equilibrated with buffer consisting of 50 m*M* Tris–HCl, 50 m*M* NaCl pH 8.0. The column was calibrated with a 12–200 kDa molecular-weight kit for gel-filtration chromatography (Sigma–Aldrich). To determine the elution volume of each protein in the kit and of *Se*EN, the protein standards were dissolved in equilibration buffer to a final concentration of 1 mg ml^−1^ and *Se*EN was diluted to 1 mg ml^−1^. 100 µl of each protein was injected into the column. The size of purified *Se*EN was then estimated through interpolation in the calibration curve.

### 2.3. Steady-state kinetic assays

The specific activity of *Se*EN for the dehydration of 2-phospho-d-glycerate (2PGA) was followed by coupling the production of phosphoenolpyruvate (PEP) to the oxidation of NADH using pyruvate kinase (PyrKin) and alanine dehydrogenase (ALDH). In this assay, the enolase dehydrates 2PGA to PEP, which is used to phosphorylate ADP to ATP using PyrKin, producing pyruvate. Next, pyruvate is reduced to alanine by ALDH using NADH, the concentration decay of which is followed spectrophotometrically at 340 nm using a Synergy H4 hybrid microplate reader (BioTek Instruments). The standard assay consisted of 100 m*M* HEPES pH 8.0, 100 m*M* KCl, 1.0 m*M* MgCl_2_, 1.2 m*M* ADP, 54 µg ml^−1^ ALDH, 7.5 µg ml^−1^ PyrKin, 0.64 m*M* NADH, 1.0 µg ml^−1^ *Se*EN and 2PGA in a final volume of 250 µl at 30°C. The steady-state kinetic parameters, *k*_cat_ and *K*_m_, were determined by the initial rates method, fitting velocity data to the standard Michaelis–Menten equation.

### 2.4. Crystallization

Crystals of *Se*EN were grown using the sitting-drop vapour-diffusion method in 96-3 IntelliPlate trays (Art Robbins Instruments; Table 2 ▸). Drops consisted of 1.0 µl of a 1:1 mixture of protein sample (25 mg ml^−1^ in 10 m*M* HEPES pH 8, 20 m*M* NaCl) and reservoir solution prepared with an Oryx8 liquid pipettor robot (Douglas Instruments). Initial hits appeared after 3–4 days of incubation at 20°C in a medium consisting of 0.1 *M* HEPES pH 7.5, 10–15%(*w*/*v*) PEG 200, 0.15–0.2 *M* calcium acetate (mother liquor). Crystals were mounted in nylon loops (Hampton Research) and flash-cooled in liquid nitrogen using 7 *M* sodium formate pH 7.0 as a cryoprotectant. In order to obtain the complex with PEP, crystals were soaked in a solution of mother liquor with 50 m*M* PEP at pH 7.4 for 30 min, cryoprotected in 7 *M* sodium formate at pH 7.0 and flash-cooled in liquid nitrogen.

### 2.5. Data collection, processing and structure solution

Diffraction data for apo *Se*EN were collected remotely on beamline BL7-1 at Stanford Synchrotron Radiation Light Source (SSRL) and data for the PEP complex were collected on beamline 08ID-1 at the Canadian Light Source (CLS) (Table 3 ▸). Reflections were indexed and integrated with *XDS* (Kabsch, 2010 ▸) and *iMosflm* (Battye *et al.*, 2011 ▸), while data scaling and truncation was conducted with *AIMLESS* (Evans & Murshudov, 2013 ▸). The structure was solved by molecular replacement using *Phaser* (McCoy *et al.*, 2007 ▸). The structure of enolase from *E. coli* (PDB entry 1e9i; Kühnel & Luisi, 2001 ▸), which shares 62% sequence identity with *Se*EN, was used as a search model, where all noncovalently bound ligands, alternate conformers and solvent molecules were removed and a random shift of 0.3 Å was applied to the remaining protein atom coordinates to minimize model bias. Structure refinement was performed with *REFMAC*5 (Murshudov *et al.*, 2011 ▸) and the *CCP*4 suite of programs (Winn *et al.*, 2011 ▸) (Table 4 ▸). Initial density maps were used for automated building with *Buccaneer* (Cowtan, 2006 ▸) followed by manual building with *Coot* (Emsley *et al.*, 2010 ▸), using σ_A_-weighted 2*F*_o_ − *F*_c_ and *F*_o_ − *F*_c_ Fourier difference maps. Structure validation was performed with the PDB Validation Service (Westbrook *et al.*, 2003 ▸) and the built-in functions in *Coot*. Figures were prepared with *PyMOL* 2.3.0 (Schrödinger) and *CorelDRAW* X7 (Corel). Metal-ion sites were checked with *CMM* (Handing *et al.*, 2018 ▸). Real-space correlation coefficients were calculated with *MolProbity* (Williams *et al.*, 2018 ▸).

### 2.6. Bioinformatic analysis

The construction of sequence-similarity networks (SSNs) was conducted with the *EFI-EST* server (Gerlt *et al.*, 2015 ▸) for Pfam (Mistry *et al.*, 2021 ▸) families PF03952 (59 600 sequences) and PF00113 (55 120 sequences), which correspond to the N-terminal capping domains and C-terminal TIM-barrel domains of enolases, respectively. Edges were drawn for node pairs sharing a *BLAST* alignment −log(*E*-score) of 100 or better, which corresponds to a sequence identity of approximately 50% or higher. The SSN size was further simplified by sampling the UniRef90 database, grouping together nodes sharing up to 40% sequence identity into representative nodes (rep nodes). Finally, an SSN with 1231 rep nodes and 46 008 edges was obtained for the enolase family. *Cytoscape* 3.8.2 (Shannon *et al.*, 2003 ▸) was utilized for network analysis, layout calculation and the evaluation of neighbourhood connectivity distribution (González, 2021 ▸). The oligomeric state of selected PDB enolase structures was estimated taking into account the buried molecular surface area (BSA) calculated with the *PISA* server (Krissinel & Henrick, 2007 ▸) (Supplementary Table S1). In order to analyse the enolase sequences with known experimentally determined structures that were present in the SSN, a subnetwork was constructed with those rep nodes containing at least one entry with an associated PDB entry, resulting in a network with 18 rep nodes and 101 edges (Supplementary Fig. S1), comprising 89 PDB entries mapped to 32 UniProt entries (Supplementary Table S2). These 32 enolase sequences were aligned with the online version of *MAFFT* (Katoh *et al.*, 2019 ▸) and a 1000-bootstrap neighbour-joining unrooted cladogram was also obtained with the server, considering only 333 conserved positions in an alignment of 556 positions and 406 gap-free sites, using the JTT substitution model. *FigTree* 1.4.4 (http://tree.bio.ed.ac.uk/software/figtree/) was used for tree display and *CorelDRAW* (Corel) was used for figure preparation.

## 3. Results and discussion

*Se*EN was purified to homogeneity, as shown by a single band on SDS–PAGE immediately above that of carbonic anhydrase (45.7 kDa) from the Kaleidoscope pre-stained standards, in line with the theoretical value of 46.7 kDa for histidine-tagged *Se*EN. The kinetic parameters for dehydration of 2PGA were estimated using a coupled system with PyrKin and ALDH. Briefly, after dehydration of 2PGA to PEP, pyruvate is both produced from PEP and reduced to alanine, leading to NADH consumption, which is followed spectrophotometrically at 340 nm. Using this setup, a Michaelis constant *K*_m_ of 61 ± 4 µ*M* and a *k*_cat_ of 7.4 ± 0.1 s^−1^ (*k*_cat_/*K*_m_ = 1.2 × 10^5^ *M* ^−1^ s^−1^) were obtained for the 2PGA dehydration reaction of *Se*EN. Such a catalytic efficiency value is comparable with available parameters for enolases (Table 5 ▸).

Crystals of recombinant *Se*EN were grown in a medium consisting of 0.1 *M* HEPES, 0.2 *M* calcium acetate, 15%(*w*/*v*) PEG 200 pH 7.5 (Table 2 ▸, Supplementary Fig. S2). Apo *Se*EN crystals belonged to the body-centred tetragonal space group *I*422, with one monomer per asymmetric unit; soaking with PEP changed to the space group to primitive tetragonal, *P*42_1_2, with a dimer in the asymmetric unit, in which each monomer was refined with a single molecule of PEP. X-ray diffraction data-collection and refinement statistics are summarized in Tables 3 ▸ and 4 ▸. In general, the most significant conformational differences were observed in loop 43–51 (PSGASTGTF), which adopts an open (apo) or closed (PEP-bound) form, with an root-mean-square deviation (r.m.s.d.) of 0.221 Å for the fitting of 2519 atoms between the two forms. Additional subtle conformational changes occur upon substrate binding, leading to a shift in the space group from *I*422 (one polypeptide chain per asymmetric unit) to *P*42_1_2 (two polypeptide chains per asymmetric unit), with an r.m.s.d. of 0.074 Å for the fitting of 2567 atoms between chains.

**Figure 1:**
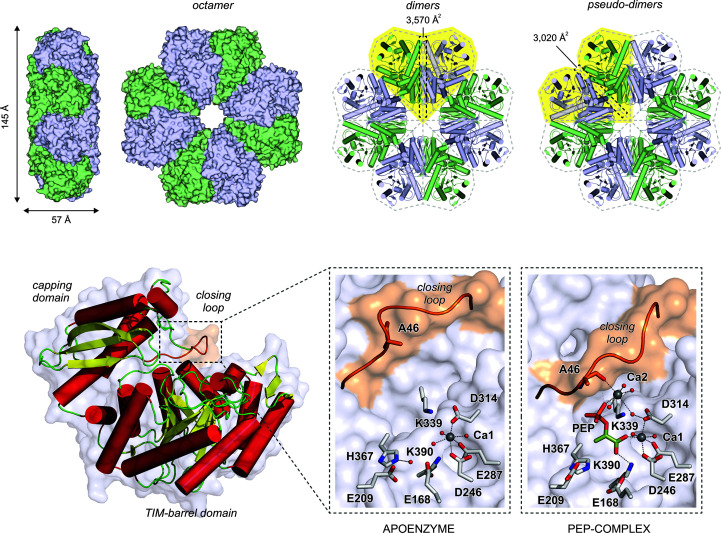
Overall crystal structure of *Se*EN. The enzyme is likely to adopt an octameric quaternary structure comprising four heart-shaped dimers or pseudo-dimers (shaded yellow). In the resting state, *Se*EN shows one Ca^2+^ ion (Ca1 site) coordinated to Asp246, Glu287 and Asp314. The segment 43-PSGASTGTF-51 is conformationally flexible and closes on the active-site cleft upon sequential entrance of the substrate and a second Ca^2+^ ion (Ca2 site). In this way, the backbone carbonyl of Ala46 becomes a ligand of Ca2. Proton extraction from 2PGA is assumed to occur from behind by Lys339 acting as a strong base, whereas hydration of PEP is believed to take place from the opposite side by a water molecule hydrogen-bonded to Glu209 and Glu168.

*Se*EN displays the typical structural features of this enzyme family. The enzyme monomer comprises the customary C-terminal TIM-barrel and N-terminal capping domains, with a metal-binding active site located between them (Fig. 1 ▸). An overall fold inspection of *Se*EN readily shows the characteristic unusual α_2_β_2_(αβ)_6_ topology, a variant of the symmetric (αβ)_8_-barrel TIM fold, which was witnessed early on for the yeast enzyme, which was the first enolase crystal structure to be determined (Lebioda *et al.*, 1989 ▸). Buried surface area (BSA) calculations allow us to estimate that *Se*EN can adopt at least an octameric quaternary structure (Supplementary Table S1). Enolases generally adopt a dimeric or octameric structure, although some exist in solution as a mixture of monomers, dimers and octamers, depending on the buffer composition, as reported for *Helicobacter pylori* enolase (López-López *et al.*, 2018 ▸).

Indeed, analysis of *Se*EN with *PISA* indicates that the octamers are stabilized by two kinds of contacts, which we refer to here as ‘main contacts’ and ‘pseudo-contacts’ (Fig. 1 ▸). Main contacts display a BSA of 3570 Å^2^ and define the heart-shaped dimers typical of dimeric enolases. On the other hand, pseudo-contacts exhibit a BSA of 3020 Å^2^ and define pseudo-dimers which lead to the formation of octamers in combination with the main contacts. Assuming that the strength of a given contact is proportional to the BSA between interacting monomers, we anticipate that *Se*EN would also exhibit a dynamical equilibrium between monomers, dimers and octamers comparable with the scenario in *H. pylori* enolase, since the BSA for contacts in dimers is only 18% larger than the BSA for pseudo-contacts in pseudo-dimers, *i.e.* pseudo-contacts are marginally weaker than main contacts. In fact, during purification by gel filtration *Se*EN showed a major elution peak corresponding to the monomeric form, suggesting that oligomerization *in vivo* requires specific conditions.

**Figure 2:**
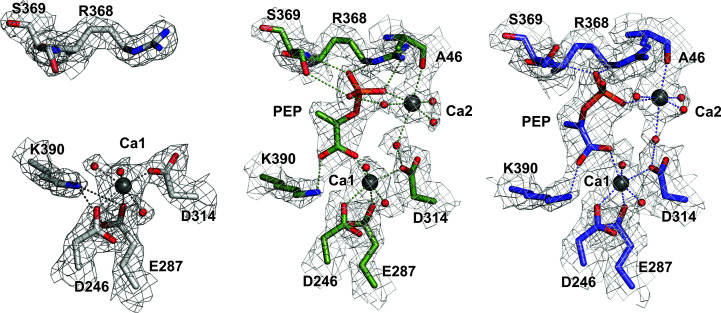
Electron-density distribution around the ligand-binding sites of apo *Se*EN (PDB entry 4rop) (*a*) and PEP-bound *Se*EN (PDB entry 5j04) chains *A* and *B* (*b*, *c*). For simplicity, only those residues that directly interact (dotted lines) with the Ca^2+^ ions and PEP are displayed. Wireframe surfaces indicate 2*F*_o_ − *F*_c_ electron-density maps contoured at 1.5σ. Grey spheres indicate the Ca1 and Ca2 metal-binding sites. Red spheres indicate water molecules.

Diffracting crystals of *Se*EN were obtained in the as-purified apo form (PDB entry 4rop) and in complex with PEP (PDB entry 5j04), allowing an analysis of the structural changes that take place upon substrate binding. Considering that both crystal forms grow in a mother liquor containing 150–200 m*M* calcium acetate, metals were refined as Ca^2+^ ions, even though the available literature indicates that enolases generally contain Mg^2+^ ions as cofactors. Nevertheless, taking into account the chemical similarity between Ca^2+^ and Mg^2+^ ions, it is likely that both metals have comparable structural effects. In the resting state of the enzyme, a ‘catalytic’ Ca^2+^ ion appears to be bound at the bottom of a wide cleft (Ca1 site), coordinated to Asp246, Glu287 and Asp314. Crystal soaking with PEP leads to binding of a second metal ion (Ca2 site), which partly stabilizes the negative charge of the PEP phosphate moiety while anchoring the flexible loop in the closed conformation and is coordinated to the Ala46 carbonyl. The second metal ion has been termed ‘structural’ since it aids in stabilizing the closed loop through its interaction with specific residues (the carbonyl of Ala46 in the loop PSGASTGTF) and the phosphate moiety of PEP (Fig. 2 ▸). In addition, the negatively charged phosphate group in PEP appears to form a salt bridge to the guanidinium side chain of Arg368 and a hydrogen bond to the backbone and side chain of Ser369, further stabilizing the ligand and giving rise to an intricate hydrogen-bond network around it. On comparing chains *A* and *B* of the PEP-complex structure, bound PEP appears to exhibit some conformational freedom, which is accompanied by hydrogen bonds to Arg368 and Ser369.

**Figure 3:**
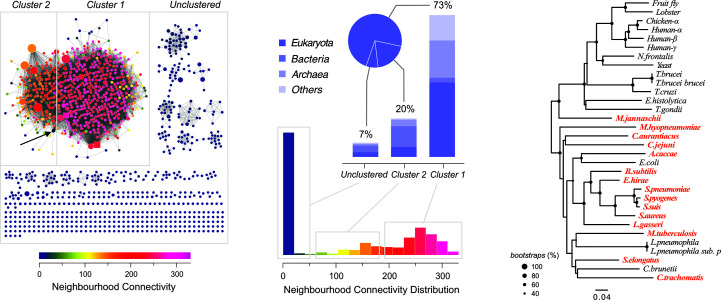
(*a*) Sequence-similarity network (SSN) of all enolase sequences currently available in the UniProt database (1231 rep nodes; 46 008 edges). Edges connecting nodes indicate at least 50% sequence similarity. Node size is proportional to the number of IDs per rep node, whereas square rep nodes indicate the presence of at least one experimentally determined enolase structure (*i.e.* associated PDB entries). The node containing the *Se*EN sequence is indicated by a black arrow. (*b*) The NC distribution indicates that all functionally and structurally characterized enolases belong to the largest cluster, accounting for 73% of the family, of which about 50% is composed of eukaryotic enolases. (*c*) Unrooted phylogenetic tree of 32 structurally characterized enolases (see Supplementary Table S1). Organism names highlighted in orange indicate enolase structures that are predicted to exhibit an octameric quaternary structure.

In order to evaluate the context in which *Se*EN is located within the enolase family of proteins, a sequence-similarity network (SSN) was constructed with all enolase sequences in the UniProt database using the *EFI-EST* server (Gerlt *et al.*, 2015 ▸). Currently, the enolase family comprises more than 100 000 sequences (Pfam families PF03952 and PF00113); therefore, SSN analysis is a convenient approach to explore such a large data set. As described in Section 2, an SSN with 1231 rep nodes and 46 008 edges was obtained for the enolase family. SSN neighbourhood connectivity (NC) reveals a major connected component with two clusters, averaging about 150 and 250 neighbours per node, and a residual group of unclustered sequences (Fig. 3 ▸). Considering that a sequence similarity of approximately 50% or higher was assumed to draw edges, a connected component indicates a group of sequences that share this similarity cutoff. The higher the NC value, the higher the shared sequence similarity; therefore, NC values define functionally analogous families. About 73% of all enolases belong to the major cluster, 20% to a less interconnected cluster and 7% remain unclustered. Notably, all enolases functionally and structurally characterized thus far belong to the major cluster, while the cyanobacterial *Se*EN reported here is borderline between clusters 1 and 2. In other words, 27% of the sequence space of all enolases remains experimentally unexplored. The 89 enolase structures currently available in the Protein Data Bank can be mapped to 32 UniProt entries (Supplementary Table S2). A dendrogram based on a sequence alignment of such sequences places *Se*EN in a clade of prokaryotic, typically octameric enolases (Fig. 3 ▸). This observation is consistent with BSA analysis of the crystal lattice molecular packing. Even though one or two molecules per asymmetric unit are found in the structures of *Se*EN in the apo and PEP-bound forms, respectively, both structures indicate that *Se*EN is able to adopt an octameric quaternary structure. Nevertheless, it is worth noting that the enolase sequences might not reflect solely their catalytic performance in terms of dehydration of 2PGA or their ability to adopt octameric structures, but also additional functions unrelated to dehydration of 2PGA. In fact, these enzymes belong to a group of ancient proteins that have evolved so-called moonlighting functions, participating in broad protein–protein interaction networks inside and outside the cell (Paludo *et al.*, 2015 ▸).

**Figure 4:**
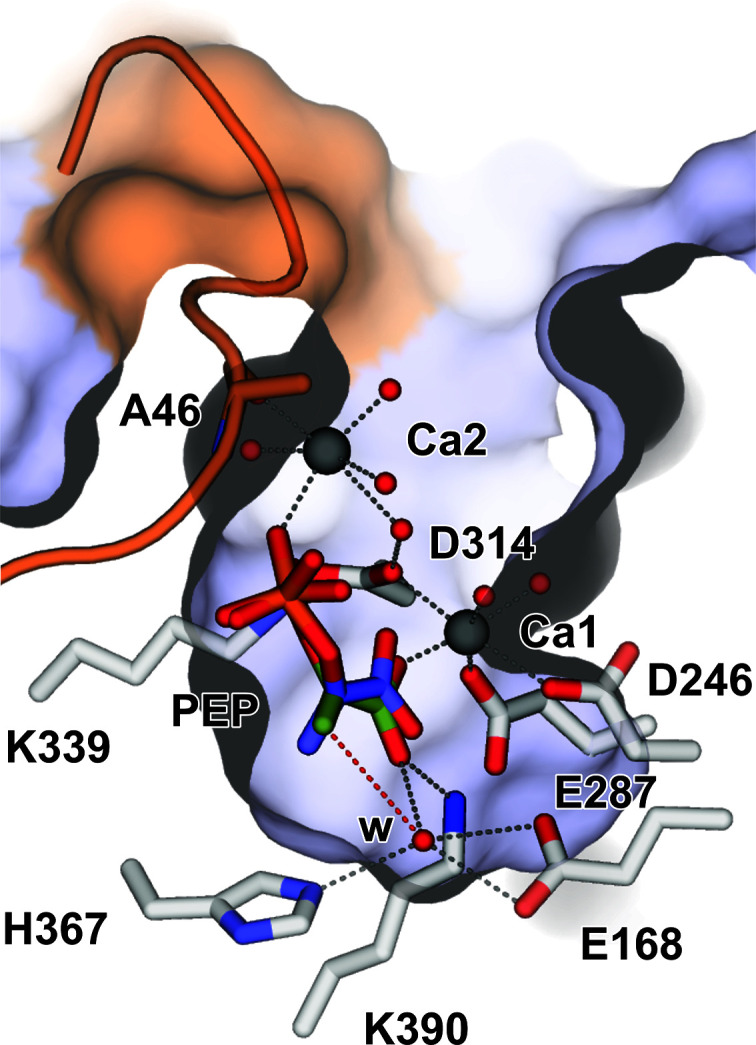
Binding of PEP in the active site of *Se*EN. The PEP molecule is shown in the two conformations found in chains *A* (green) and *B* (blue) of PDB entry 5j04. The water molecule ‘w’ is 3.5 Å from C3 of the PEP molecule (red dotted line) and is hydrogen-bonded to His367 and Glu168. Compare with Fig. 2 ▸. Presumably, during the dehydration of 2PGA or the hydration of PEP (bottom), a water molecule is exchanged from the Glu168 side, whereas proton extraction occurs from the opposite side, with Lys339 acting as a strong base.

The enolase reaction mechanism has been extensively studied, particularly in the yeast enzyme (Reed *et al.*, 1996 ▸), and shows remarkable mechanistic similarities to muconate-lactonizing enzyme and mandelate racemase (Neidhart *et al.*, 1990 ▸; Gerlt *et al.*, 2005 ▸, 2012 ▸). Their catalytic mechanism is defined by a partial reaction in which an α-proton of a substrate carboxylic acid is removed by a nearby strong base, forming a C1=C2 double bond, with the additional negative charge developing in the carboxylate moiety stabilized by a metal ion such as Mg^2+^ (Fig. 4 ▸). In this way, the α-carbon becomes an electron-rich species that reacts with another proton in a second stage. In enolases, a β-elimination takes place, in which a β-OH is lost as a water molecule and an α–β (C2=C3) double bond is formed. These characteristic reaction steps also take place within a conserved protein scaffold. The capping domain, including its flexible loop, defines the volume, shape and polarity of the active site, thereby selecting the substrate that is presented to the catalytic residues. A reaction mechanism for *Se*EN can be pictured considering the proposed reaction mechanism of yeast enolase. Binding of PEP at the bottom of the cleft defines two sides: an inner solvent-inaccessible side, where proton extraction by Lys339 acting as a strong base would take place, and a solvent-accessible side, from where PEP hydration is believed to occur through activation by a hydrogen-bond network involving Glu168 and His367.

Production of value-added biomass using photosynthetic microbes such as algae and cyanobacteria has been proposed as a cost-effective and environment-friendly alternative to biomass from fossil fuels (Claassens *et al.*, 2016 ▸; Quintana *et al.*, 2011 ▸; Scott *et al.*, 2010 ▸). The results presented here expand our comprehension of cyanobacterial metabolism, enlarging our toolbox of enzymes for metabolic engineering applications.

## Supplementary Material

PDB reference: enolase from *Synechococcus elongatus*, 4rop

PDB reference: complex with phosphoenolpyruvate, 5j04

Supplementary Figures and Tables. DOI: 10.1107/S2053230X22003612/us5142sup1.pdf

## Figures and Tables

**Table 1:** Macromolecule-production information

Source organism	*Synechococcus elongatus* PCC7942
DNA source	Synthetic
Expression vector	pET-42a
Expression host	*Escherichia coli* Arctic Express (DE3)
Primers	Cloning into NdeI and XhoI restriction sites of vector pET-42a
Complete amino-acid sequence of the construct produced	MPDDYGTQIAEITAREILDSRGRPTVEAEVHLEDGSVGLAQVPSGASTGTFEAHELRDDDPSRYGGKGVQKAVENVSAIEDALIGLSALDQEGLDKAMIALDGTPNKKNLGANAILAVSLATAHAAATSLNLPLYRYLGGPLANVLPVPMMNVINGGAHADNNVDFQEFMIMPVGAPSFKEALRWGAEVFHALAKVLKDKGLATGVGDEGGFAPNLGSNKEALELLLTAIEAAGYKPGEQVALAMDVASSEFYKNGLYTCDGVSHEPAGMIGILADLVSQYPIVSIEDGLQEDDWSNWKTLTQQLGSTVQLVGDDLFVTNPDRLQSGIEQGVGNAVLIKLNQIGTLTETLRTIDLATRSGYRSVISHRSGETEDTTIADLAVATRAGQIKTGSLSRSERIAKYNRLLRIEAALGENALYAGAIGLGPKGRLEHHHHHHHH

**Table 2:** Crystallization

Method	Sitting drop
Plate type	96-3 IntelliPlate
Temperature (K)	293
Protein concentration (mg ml^−1^)	25
Buffer composition of protein solution	10 m*M* HEPES pH 8, 20 m*M* NaCl
Composition of reservoir solution	0.1 *M* HEPES pH 7.5, 10–15%(*w*/*v*) PEG 200, 0.15–0.2 *M* calcium acetate
Volume and ratio of drop	1 µl, 1:1
Volume of reservoir (ml)	0.1

**Table 3:** Data collection and processing Values in parentheses are for the highest resolution shell.

	Apoenzyme	PEP complex
Diffraction source	BL7-1, SSRL	08ID-1, CLS
Wavelength (Å)	1.12709	0.97949
Resolution range (Å)	37.7–2.05 (2.10–2.05)	82.1–2.30 (2.38–2.30)
Total No. of reflections	328759 (25708)	406551 (39756)
No. of unique reflections	30631 (2261)	44630 (4315)
Completeness (%)	98.7 (99.9)	99.8 (100)
Temperature (K)	100	100
Detector	ADSC Quantum 315r CCD	Rayonix MX300 CCD
Space group	*I*422	*P*42_1_2
*a*, *b*, *c* (Å)	164.3, 164.3, 75.5	164.2, 164.2, 72.7
α, β, γ (°)	90, 90, 90	90, 90, 90
Mosaicity (°)	0.37	0.66
*R*_merge_	0.115 (0.631)	0.196 (0.829)
*R*_meas_	0.121 (0.664)	0.208 (0.878)
*R*_p.i.m._	0.038 (0.207)	0.068 (0.285)
Multiplicity	10.1 (10.2)	9.1 (9.2)
〈*I*/σ(*I*)〉	9.0 (2.2)	8.7 (3.1)
CC_1/2_	0.998 (0.928)	0.992 (0.919)
Overall *B* factor from Wilson plot (Å^2^)	34.3	24.3

**Table 4:** Structure solution and refinement Values in parentheses are for the highest resolution shell.

	Apoenzyme	PEP complex
PDB code	4rop	5j04
Resolution range (Å)	37.7–2.05 (2.10–2.05)	82.1–2.30 (2.38–2.30)
No. of reflections, working set	30631	42379
No. of reflections, test set	1561 [4.8%]	2189 [4.9%]
Final *R*_cryst_ (%)	20.9 (29.2)	21.8 (24.4)
Final *R*_free_ (%)	23.3 (33.3)	24.3 (29.2)
Chains	1	2
No. of non-H atoms		
Protein	3144	6278
Ligand	4 [Ca^2+^]	35 [Ca^2+^, ACT, PEP]
Water	154	191
Total	3302	6504
R.m.s. deviations		
Bonds (Å)	0.015	0.015
Angles (°)	1.92	1.91
Average *B* factor (Å^2^)	38.7	32.3
Ramachandran favoured	409 [96.4%]	830 [97.2%]
Ramachandran allowed	12 [3.32%]	12 [2.61%]
Ramachandran outliers	1 [0.24%]	2 [0.24%]
Real-space correlation coefficients		
PEP	—	0.95 [*A*], 0.93 [*B*]
Ca^2+^, site Ca1	0.99	0.96 [*A* and *B*]
Ca^2+^, site Ca2	—	0.94 [*A*], 0.83 [*B*]

**Table 5:** Kinetic parameters of *Se*EN as obtained in this work, compared with those of selected enolases

Enzyme organism	*k*_cat_ (s^−1^)	*K*_m_ (µ*M*)	*k*_cat_/*K*_m_ (*M* ^−1^ s^−1^)	Reference
Yeast	78	300	2.6 × 10^5^	Reed *et al.* (1996 ▸)
*Klebsiella pneumoniae*	52.7	425	1.2 × 10^5^	Pietkiewicz *et al.* (2009 ▸)
Human β-enolase	81.7	199	4.1 × 10^5^	Pietkiewicz *et al.* (2009 ▸)
*Staphylococcus aureus*	830	365	2.3 × 10^5^	Wu *et al.* (2015 ▸)
*S. elongatus*	7.4	61	1.2 × 10^5^	This work
