# Supplementary material for: The structure of Synechococcus elongatus enolase reveals key aspects of phosphoenolpyruvate binding
Source: Acta Crystallogr F Struct Biol Commun. 2022 Apr 3;78(Pt 4):177–84. doi: 10.1107/S2053230X22003612 (PMC8996150; doi:10.1107/S2053230X22003612)
Supplement: Supplementary file 1 [file f-78-00177-sup1.pdf]

STRUCTURAL BIOLOGY COMMUNICATIONS

Volume 78 (2022)

Supporting information for article:

The structure of *Synechococcus elongatus* enolase reveals key aspects of phosphoenolpyruvate binding

Javier M. González, Ricardo Martí-Arbona, Julian C.-H. Chen and Clifford J. Unkefer

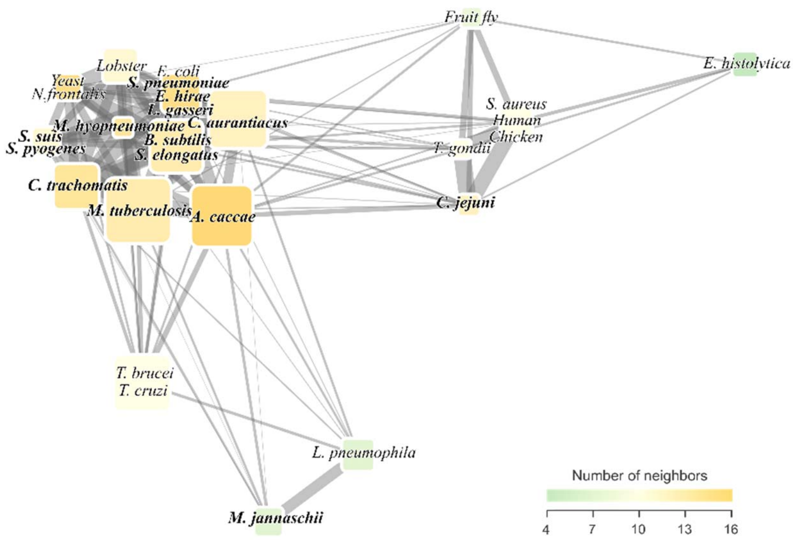

Figure S1 Sequence similarity subnetwork (18 rep nodes by 101 edges) comprising those rep nodes and edges in the family-wide SSN including at least one enolase sequence with known PDB structure. Colouring according to number of neighbours (also known as node degree) and edge thickness proportional to sequence similarity allow a better visualization of clusters in the network. Compare with the dendrogram in Figure 3.

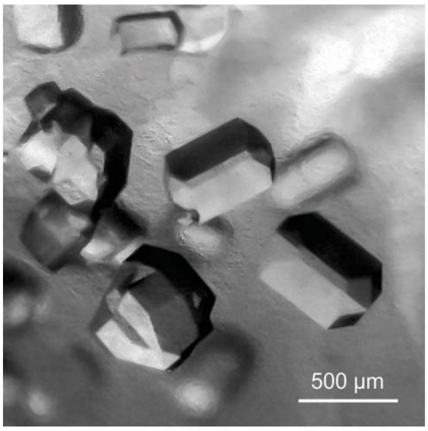

Figure S2 *Se*EN crystals.

Table S1 Buried area (in Å^2) of selected enolase crystallographic structures for quaternary structure estimation, as calculated with the PISA server (Krissinel & Henrick, 2007).

Organism	UniProt	PDB	Dimer	Octamer
Trypanosoma cruzi	Q4DZ98	4G7F	0	0
Coxiella burnetii	Q83B44	3TQP	3,760	0
Toxoplasma gondii	B9PH47	3OTR	3,770	0
Escherichia coli (strain K12)	P0A6P9	1E9I	4,060	0
Legionella pneumophila	A0A2S6F4U1	6NBM	4,260	0
Homarus gammarus (European lobster)	P09104	1PDY	4,530	0
Human γ-enolase	Q97QS2	1TE6	4,820	0
Saccharomyces cerevisiae (Baker's yeast)	P00924	1EBG	4,920	0
Human α-enolase	Q38BV6	2PSN	4,960	0
Trypanosoma brucei brucei	Q9NDH8	2PTZ	5,670	0
Trypanosoma brucei brucei	P56252	2PU1	5,780	0
Entamoeba histolytica	P51555	3QTP	5,920	0
Legionella pneumophila sub. pneumophila	Q5ZTX1	6NB2	6,130	0
Drosophila melanogaster (Fruit fly)	P15007	5WRO	6,920	0
Human β-enolase	P13929	2XSX	8,670	0
Streptococcus suis	K7N5M7	4EWJ	3,350	22,870
Lactobacillus gasseri	Q042F4	4MKS	3,410	23,150
Chloroflexus aurantiacus	A9WCM4	4YWS	3,290	23,630
Synechococcus elongatus	Q5N3P4	4ROP	3,570	23,920
Streptococcus pyogenes	Q5XD01	3ZLH	3,650	24,770
Streptococcus pneumoniae	Q60173	1W6T	4,190	26,920
Anaerostipes caccae	B0MAG5	3UJ2	4,810	26,960
Bacillus subtilis (strain 168)	P37869	4A3R	3,480	27,120
Campylobacter jejuni	P42448	3QN3	4,750	29,170
Staphylococcus aureus	O69174	5BOE	5,270	31,760
Enterococcus hirae	Q8GR70	1IYX	4,780	32,180
Mycobacterium tuberculosis	P9WNL1	6L7D	5,380	32,670
Mycoplasma hyopneumoniae	A0A223MA21	6J36	5,840	33,530
Methanocaldococcus jannaschii	P06733	2PA6	4,310	34,170
Chlamydia trachomatis	B0BA40	6O4N	5,670	38,850

Table S2 PDB and associated UniProt entries present in the enolase SSN (Figure 3).

**Organism**	**PDB**	**Entry**
Anaerostipes caccae	3UJ2	B0MAG5
Bacillus subtilis (strain 168)	4A3R	P37869
Campylobacter jejuni	3QN3	P42448
Chlamydia trachomatis	6O4N	B0BA40
Chloroflexus aurantiacus	4YWS, 4Z17, 4Z1Y	A9WCM4
Coxiella burnetii	3TQP	Q83B44
Drosophila melanogaster (Fruit fly)	5WRO	P15007
Entamoeba histolytica	3QTP	P51555
Enterococcus hirae	1IYX	Q8GR70
Escherichia coli (strain K12)	1E9I, 2FYM, 3H8A, 5OHG, 6BFY, 6BFZ, 6D3Q, 6NPF	P0A6P9
Gallus gallus (Chicken)	2GUA	P51913
Homarus gammarus (European lobster)	1PDY, 1PDZ	P56252
Human α-enolase	2PSN, 3B97, 5JLZ, 5LAX, 5NI9, 5NIG, 5OCK	P06733
Human β-enolase	2XSX	P13929
Human γ-enolase	1TE6, 2AKM, 2AKZ, 3UCC, 3UCD, 3UJE, 3UJF, 3UJR, 3UJS, 4ZA0, 4ZCW, 5EU9, 5IDZ, 5TD9, 5TIJ	P09104
Lactobacillus gasseri	4MKS	Q042F4
Legionella pneumophila	6NBM	A0A2S6F4U1
Legionella pneumophila sub. pneumophila	6NB2	Q5ZTX1
Methanocaldococcus jannaschii	2PA6	Q60173
Mycobacterium tuberculosis	6L7D	P9WNL1
Mycoplasma hyopneumoniae	6J36	A0A223MA21
Neocallimastix frontalis (Rumen fungus)	2GE6	P42894
Saccharomyces cerevisiae (Baker's yeast)	1EBG, 1EBH, 1ELS, 1L8P, 1NEL, 1ONE, 1P43, 1P48, 2AL1, 2AL2, 2ONE, 2XGZ, 2XH0, 2XH2, 2XH4, 2XH7, 3ENL, 4ENL, 5ENL, 6ENL, 7ENL	P00924
Staphylococcus aureus	5BOE, 5BOF	O69174
Streptococcus pneumoniae	1W6T	Q97QS2
Streptococcus pyogenes	3ZLF, 3ZLG, 3ZLH	Q5XD01
Streptococcus suis	4EWJ	K7N5M7
Synechococcus elongatus	4ROP, 5J04	Q5N3P4
Toxoplasma gondii	3OTR	B9PH47
Trypanosoma brucei brucei	1OEP, 2PU1	Q9NDH8
Trypanosoma brucei brucei	2PTW, 2PTX, 2PTY, 2PTZ, 2PU0	Q38BV6
Trypanosoma cruzi	4G7F	Q4DZ98
